# The Physician’s Visiting List

**Published:** 1887-12

**Authors:** 


THE PHYSICIAN'S VISITING LIST. Thirty-seventh year, 1888. P. Blakiston Son & Co., Philadelphia, Pa.

No better evidence of the practical worth of this book can be offered than the uniform popularity it has enjoyed, notwithstanding the competition during the past few years.

